# Adolescent elbow osteochondral lesions following prior elbow fracture pinning

**DOI:** 10.1177/18632521221133814

**Published:** 2022-10-25

**Authors:** Ajay S Padaki, Sachin Allahabadi, Nirav K Pandya

**Affiliations:** 1 Department of Orthopaedic Surgery, University of California San Francisco, San Francisco, CA, USA; 2 Benioff Children’s Hospital Oakland, University of California San Francisco, Oakland, CA, USA

**Keywords:** Osteochondritis dissecans, osteochondral lesion, elbow surgery, cartilage surgery

## Abstract

**Purpose:**

Pinning of pediatric elbow fractures has been shown to be a safe procedure with a low complication profile. This study identified patients who underwent cartilage surgery for elbow osteochondral lesions or osteochondritis dissecans who had prior ipsilateral elbow pinning.

**Methods:**

Records of patients who underwent ipsilateral cartilage surgery for osteochondritis dissecans and prior percutaneous pinning for elbow fractures were identified. Demographics were compiled and the clinical, radiographic, and surgical results were tabulated for patients with at least 1-year of follow-up from initial presentation.

**Results:**

In total, 6/52 (11.5%) pediatric patients from 2012 to 2021 who underwent isolated elbow osteochondritis dissecans surgery (mean age at surgery 13.4 ± 1.5 years) had a history of ipsilateral elbow pinning (mean age at surgery 6.9 ± 2.4 years). Of these, five had a history of a supracondylar fracture while one patient sustained a lateral condyle fracture. Overall, three of six patients had mechanical symptoms at presentation and three had abnormal radiographs. All patients underwent pre-operative magnetic resonance imaging and the five patients with an osteochondritis dissecans lesion <1cm^2^ underwent arthroscopy and microfracture while one with a 4-cm^2^ lesion underwent open osteochondral allograft transfer. All patients demonstrated improved motion at final follow-up and all patients were able to return to full desired activity following surgery.

**Conclusion:**

This study demonstrates that the history of elbow fracture pinning may predispose patients to future elbow chondral injuries in adolescence. Although patients appear to do well following consequent osteochondritis dissecans surgery, patients and parents may be advised of possible association of elbow pinning and elbow osteochondral lesions.

**Level of Evidence:**

III, case–control study

## Introduction

Pediatric elbow fractures represent some of the most common injuries treated by pediatric orthopaedic surgeons with an estimated incidence of approximately 300/100,000.^1,2^ While slightly more than half of fractures recorded are supracondylar, lateral condyles comprise the second most frequent etiology at 20%–25% of all pediatric elbow fractures.^3^ Large cohort studies have demonstrated the relative safety and efficacy of closed reduction and percutaneous pinning (CRPP), with overall complications rates of 2%–4%.^4^ Superficial infections and pin migration are most frequently reported, with iatrogenic neurovascular injury reported at an incidence well below 1%.^5,6^ Recently, however, small clinical and radiological case series highlighted the potential link between prior elbow pinning and adolescent elbow osteochondritis dissecans (OCD) development.^7,8^ Specifically, the subsequent development of a fishtail deformity, or lateral trochlear hypoplasia, and OCD have been investigated.

Osteochondral lesions in the adolescent elbow are typically found on the capitellum in overhead athletes and gymnasts, and may have worse outcomes in these high upper extremity demand athletes.^9,10^ OCD lesions in adolescent baseball players were identified in 3.4% of players, though many of these patients were asymptomatic.^11^ While many osteochondral lesions can be treated nonoperatively, the presence of loose bodies, mechanical symptoms, unstable lesions, and continued symptomatology warrant surgical intervention.^12,13^ Operative treatment of pediatric elbow chondral injuries includes a diverse array of treatment options ranging from arthroscopy, microfracture, loose body removal, and chondroplasty to osteochondral autograft transfer (OATS), osteochondral allograft transfer, and autologous chondrocyte implantation (ACI) with and without a matrix.^14^ While the age of the patient, size and stability of the lesion, level of overhead activity, and surgeon preference help delineate surgical treatment, studies directly contrasting treatment arms have found similar results among operative cohorts.^15,16^

While the specific cause of elbow osteochondral lesions may be multifactorial, there is increasing interest in identifying risk factors for its development. The purpose of this study was to investigate the association between patients requiring surgery for elbow osteochondral lesions and prior supracondylar humerus or lateral condylar pinning, including evaluating patient demographics, imaging findings, and treatment outcomes.

## Materials and methods

The institutional review board (IRB) of the University of California San Francisco approved this study to retrospectively identify patients who were treated operatively for elbow OCD lesions. Patients were treated algorithmically with elbow radiographs obtained at the first visit following history and physical examination. All patients that were suspected to have OCD lesions underwent magnetic resonance imaging (MRI) to further characterize the lesion. Patients with stable lesions and without loose bodies or mechanical symptoms underwent a minimum of 3 months of nonoperative management consisting of physical therapy to restore range of motion (ROM), activity modification and throwing cessation for baseball players, and pain control. Operative treatment was reserved for patients with unstable lesions, mechanical symptoms, and those who failed conservative treatment.

All elbow OCD surgical patients of the senior author (NKP) were reviewed over a 9-year period (2012–2021). Patients during this interval with a prior history of pediatric elbow surgery with pinning were identified and further investigated. To be included, patients must have been followed for at least 12 months from initial presentation.

Further inclusion criteria consisted of having ipsilateral elbow cartilage and fracture surgery, age of 18 years or less at time of presentation, and having complete clinical and radiographic data at follow-up. Exclusion criteria included concomitant surgery at the time of chondral re-operation (i.e. ulnar osteotomy, ligamentous reconstruction), incomplete clinical or radiographic data, and follow-up of less than 12 months from initial presentation. While all patients underwent the initial elbow pinning by an outside surgeon, the corresponding operative reports were obtained to confirm the history of an ipsilateral procedure; each case utilized either 1.6 or 2.0 mm k-wires for fixation.

Routine elbow arthroscopy with partial synovectomy, loose body removal, chondroplasty, and microfracture of the osteochondral lesion was performed as first line treatment for patients with standard chondral defects. In one patient, with near complete lateral condyle osteonecrosis, an open osteochondral allograft and lysis of adhesions was performed through a lateral elbow Kocher open approach.

Data collected for identified patients included demographics, age at time of initial surgery, symptoms at time of surgery, age at time of chondral surgery, type of chondral surgery, clinical function, and radiographic findings. Operative reports and radiographic reports were assessed to further stratify these findings.

## Results

In total, 54 patients were identified undergoing surgery for elbow OCD during the study period. Of these, 2 patients were excluded for additional surgery at the time of chondral surgery. Of the remaining 52 patients undergoing isolated elbow osteochondral surgery, 6 (11.5%) were identified with prior elbow fracture pinning.

Five (83.3%) of the included six patients were male and the mean age at time of supracondylar or lateral condyle percutaneous pinning was 6.9 ± 2.4 years. One patient underwent lateral condyle open reduction and percutaneous pinning while the other five underwent supracondylar CRPP. The mean age at time of elbow cartilage surgery was 13.4 ± 1.5 years and mean 6.6 ± 1.9 years had passed between the two operations. Symptoms at presentation included stiffness, pain, locking, and inability to participate in athletics with half of all patients reporting mechanical symptoms. All patients were active in sports with one playing basketball, one playing basketball and participating in dancing, three playing baseball, and one participating in wrestling. The demographics and imaging findings of these patients are given in Table 1.

**Table 1.** Patient demographics and presentation summary.

*Variable*	Value
*Age at CRPP (years)*	6.9 ± 2.4
*Age at cartilage surgery (years)*	13.4 ± 1.5
*Male, n (%)*	5 (83.3)
*Mechanical symptoms, n (%)*	3 (50)
*Supracondylar, n (%)*	5 (83.3)
*Abnormal radiographs (%)*	3 (50.0)
*Loose bodies on MRI (%)*	4 (66.6)
*Mean OCD size (mm sq)*	125.8

CRPP: closed reduction and percutaneous pinning; MRI: magnetic resonance imaging; OCD: osteochondritis dissecans.

Six patients met inclusion criteria. Data are presented as mean ± standard deviation, *n* (%), or values.

**Figure 1.**
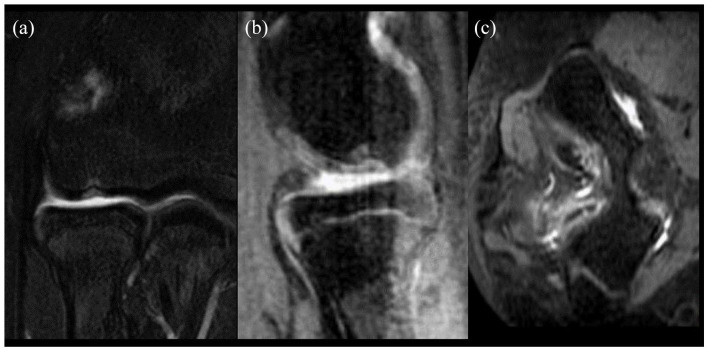
The (a) coronal, (b) sagittal, and (c) axial of a 14-year-old male patient with a 5 mm × 8 mm osteochondral capitellar defect and 9 mm × 4 mm posterior loose body.

**Figure 2.**
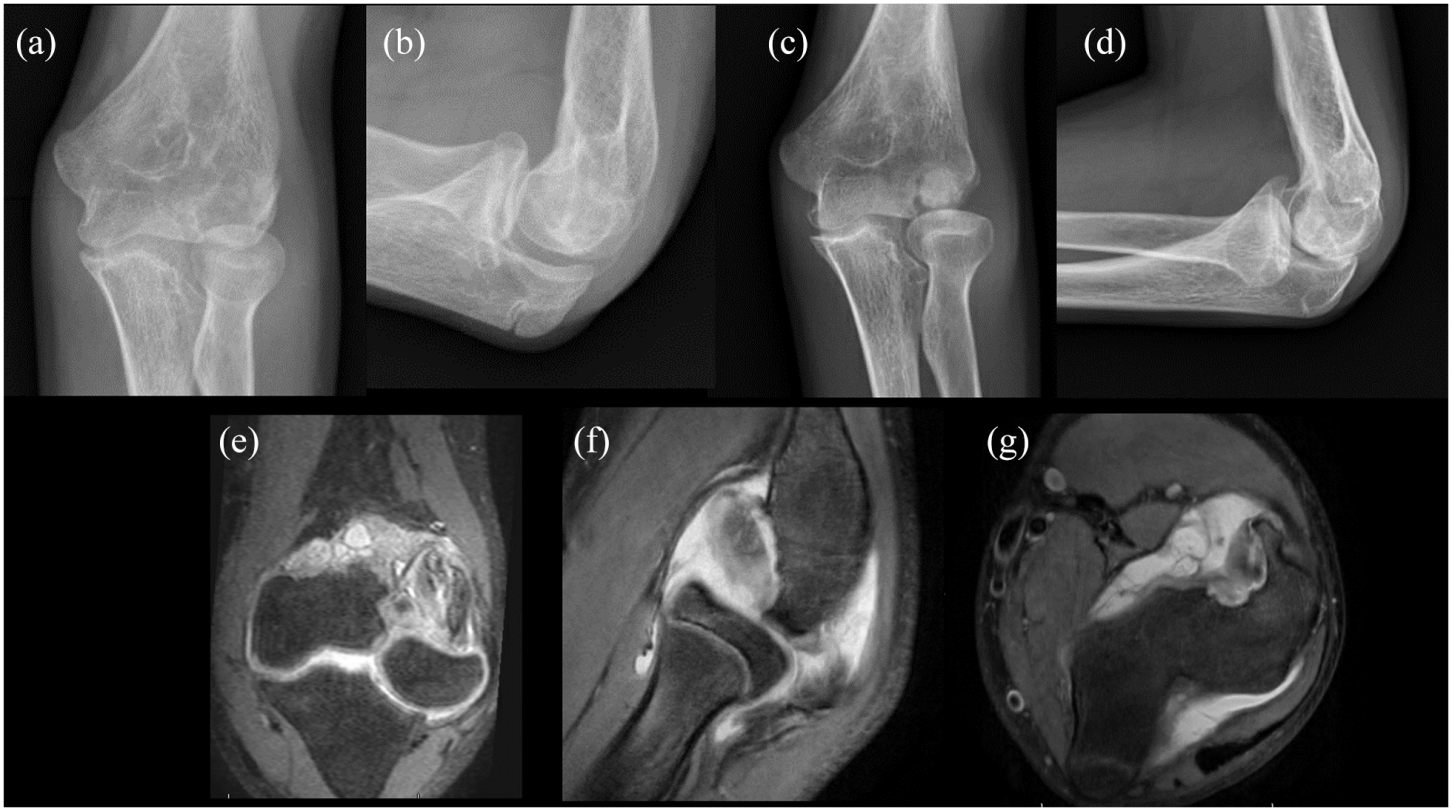
Pre- and post-operative imaging of osteochondral allograft implantation of 14-year-old male patient with a large osteochondral defect (22 mm × 22 mm). Pre-operative radiographs (a and b) and post-operative radiographs (c and d) demonstrate interval placement of an 18 mm × 18 mm osteochondral allograft. Pre-operative MRI (e, f, g) demonstrates the size of the capitellar osteochondritis dissecans.

On plain X-ray, three patients had normal findings whereas two were found to have a fishtail deformity and one was found to have radiographic evidence of a large osteochondral defect of the capitellum. All six patients had pre-operative MRI, with OCD found in the capitellum of all six patients and loose bodies found in four. A summary of these radiographic findings is shown in Table 2 and pre-operative imaging for a patient undergoing elbow arthroscopy is displayed in Figure 1. The pre-operative MRI of the patient who underwent an osteochondral allograft transfer to the defect site is displayed in Figure 2. The number of pins utilized at the index surgery ranged from 2 to 4 and all pins were laterally placed (Table 2).

**Table 2.** Pre- and post-operative patient assessments.

*Patient*	Age at cartilage surgery (year)	Capitellar OCD MRI size	OCD surgery	Pre-op ROM	Post-op ROM	Initial injury	# Pins
1	13.2	6 mm × 7 mm	Microfrx	10–130	0–140	S3	3
2	10.4	4 mm × 5 mm	Microfrx	30–130	0–140	S3*	3
3	14.4	5 mm × 8 mm	Microfrx	5–130	0–140	S3	2
4	13.4	8 mm × 9 mm	Microfrx	5–135	0–140	L^	4
5	15	8 mm × 8 mm	Microfrx	5–130	0–140	S2	3
6	14.1	22 mm × 23 mm	OCA	45–90	20–130	S3	4

OCD: osteochondritis dissecans; MRI: magnetic resonance imaging; ROM: range of motion; Microfrx: microfracture; S3: supracondylar type 3; *: dysvascular; L: lateral condyle; ^: required open reduction; S2: supracondylar type 2; OCA: osteochondral allograft.

At final follow-up, all patients demonstrated improved ROM and pain. All six patients were able to return to their desired activity level and sport. While the ROM was full in the arthroscopic cohort, a flexion contracture of 20° persisted at 12 months post-operatively in the patient who underwent open osteochondral allograft.

## Discussion

This study identified a relatively high proportion of 11.5% of children undergoing surgery for symptomatic refractory elbow OCD with history of elbow pinning mean 6.6 years prior. In this small series, five patients had prior supracondylar humerus fractures and one had a lateral condyle fracture. The five patients undergoing arthroscopic microfracture had full ROM post-operatively while the one patient undergoing osteochondral allograft transfer had a 20° flexion contracture. Despite this, all patients were able to return to desired activities after surgery. The present series suggests a link between elbow osteochondral lesions and prior elbow fracture pinning that warrants further larger scale investigation.

Osteochondral lesions of the elbow are common injuries in young athletes, generally diagnosed in adolescent throwers and gymnasts. Although many of these pediatric injuries can be treated nonoperatively, surgery has become the standard of care in patients with unstable lesions, mechanical symptoms, and those who have failed nonoperative management.^16^ While the majority of pediatric elbow OCD lesions are thought to be idiopathic, recent literature has suggested a radiographic link in patients with a history of elbow fracture pinning. The present series of patients treated by a single surgeon at a single institution suggests that >10% of patients with elbow OCD had prior pin treatment of elbow fractures. In order to validate this association, further larger scale database studies are required.

Lehnert et al.^7^ reported on seven patients with radiographic fishtail deformities with subsequent MRI confirming the presence of osteochondritis dissecans in two patients. Yamaga et al.^8^ then reported on two patients with a history of type III supracondylar fractures in whom OATS was performed harvesting cartilage from the patients’ knee. Both studies pointed to potential proximal ulnar migration causing increased force transmission through the lateral column, consequently increasing the likelihood of these patients developing OCD lesions in later years.

While literature focuses on the importance of pin placement and spread in elbow fracture fixation,^17,18^ the number of Kirschner wire passes intraoperatively needed to accomplish this is largely ignored. Despite the small diameter of the pins, numerous passage attempts could cause direct mechanical trauma to both the physis and cartilage, with sequelae that may take years to develop. It is also possible that larger pin diameters could contribute to additional chondral damage.

In addition, the heat generated during pinning may also contribute to chondral necrosis. K-wire specific investigations have demonstrated that repassage of a wire can rapidly increase the metal’s temperature to above 70°C, causing immediate cellular necrosis.^19^ Numerous studies have investigated the nature of orthopedic thermal osteonecrosis inherent to the nature of the equipment utilized.^20,21^ These risks are also conferred to pediatric patients undergoing CRPP and may also contribute to later development of chondral lesions.

The overall risk of performing CRPP in pediatric elbow fractures has been shown to be very low,^4^ with the literature largely focusing on neurovascular sequelae.^5^ This study and prior radiographic case series indicate that CRPP may also confer a low risk of these patients developing an OCD lesion, though larger studies with long-term follow-up are necessary to investigate this hypothesis. At this time, even if this risk of CRPP on the development of elbow osteochondral lesions is confirmed, it is unlikely to impact clinical follow-up for these patients. Data suggest that patients after CRPP of pediatric supracondylar humerus fractures regain ~94% of motion by 6 months follow-up with further improvement up to 1 year.^22^ After that time, it is not routine to have clinical follow-up with patients who are frequently doing well and asymptomatic. However, parents of patients could be counseled that if several years later their child is having elbow pain that they should seek early evaluation.

The major limitation of this study is the low number of patients, which prevent strong conclusions from being drawn. In addition, because none of the initial fracture pinnings were performed by the study authors, the details of the index percutaneous pinning are only captured in operative reports, with the interval between elbow pinning surgery and initial symptoms of OCD largely unmeasured. Because of the retrospective and case-control study design, only an association may be noted, and it is not possible to control for additional confounding factors such as the initial fracture or injury vs. the pin placement, activity, sport, and additional trauma patients may have sustained, among others. Furthermore, this study only evaluated patients who required surgery for elbow OCD lesions, and there are likely many with elbow OCD lesions who were treated nonoperatively who were not captured; therefore, the true rate of those with prior elbow percutaneous pinning is likely different and may be evaluated in future studies.

## Conclusion

This study demonstrates that the history of elbow fracture pinning may predispose patients to future elbow chondral injuries in adolescence. Although patients appear to do well following consequent osteochondritis dissecans surgery, patients and parents may be advised of possible association of elbow pinning and elbow osteochondral lesions.
